# Ocular involvement associated with varicella in adults

**DOI:** 10.1186/s12348-016-0117-9

**Published:** 2016-11-28

**Authors:** Salma Gargouri, Sana Khochtali, Sourour Zina, Molka Khairallah, Imen Zone-Abid, Imen Kaibi, Salim Ben Yahia, Jamel Feki, Moncef Khairallah

**Affiliations:** 1 Department of Ophthalmology, Habib Bourguiba University Hospital, Faculty of Medicine, University of Sfax, Sfax, Tunisia; 2 Department of Ophthalmology, Fattouma Bourguiba University Hospital, Faculty of Medicine, University of Monastir, Monastir, Tunisia

**Keywords:** Varicella, Uveitis, Keratitis, Acute retinal necrosis

## Abstract

**Background:**

Varicella is a common infectious disease primarily of childhood that is usually benign and self-limited. It is, however, increasingly seen in adults who are at a higher risk of severe infection. Ocular complications of varicella are relatively uncommon and have been rarely described in adults. We describe herein five adults who developed ocular involvement in association with primary varicella-zoster virus infection.

**Findings:**

Ocular manifestations included acute anterior uveitis in four eyes, with associated stromal keratitis in one of them, epithelial ulcerative keratitis in the two eyes of one patient, and acute retinal necrosis in one eye. One patient with acute anterior uveitis was treated with topical steroids and cycloplegic agents. The four other patients received topical or systemic antiviral drugs, with subsequent resolution of acute ocular inflammatory disease.

**Conclusions:**

The spectrum of chickenpox-associated ocular complications in adults is wide. Early diagnosis and appropriate management are mandatory to improve visual outcome.

## Findings

### Introduction

Primary varicella infection, also called chickenpox, is a common benign illness caused by varicella-zoster virus (VZV), typically associated with fever and characteristic exanthematous vesicular skin rash. It usually affects children, but the condition may also occur in immunocompromised or otherwise healthy adults [1].

Ocular complications associated with primary VZV infection can occur and may involve any part of the eye from the conjunctiva to the optic nerve. Data on ocular involvement associated with varicella in adults are scarce, and most publications consisted of single case reports [2–7].

We describe herein five adults who developed ocular involvement in association with varicella.

### Case reports

Demographic characteristics, clinical features, treatment modalities, and outcome in this case series of adult patients with chickenpox-related ocular involvement are summarized in Table 1. Of our five patients, four were male and one was female. The mean age was 37.8 (range, 33–42). Four were immunocompetent and one was immunocompromised. Ocular involvement was unilateral in three patients and bilateral in two patients. Ocular findings included acute anterior uveitis in four eyes, with associated stromal keratitis in one eye, epithelial keratitis in the two eyes of one patient, and acute retinal necrosis (ARN) syndrome in one eye.

**Table 1:** Clinical features, treatment modalities, and outcome in adults with chickenpox-associated ocular complications

Case	Gender/age (years)	Past medical history	Time from skin eruption to ocular symptoms (weeks)	Affected eye	Initial visual acuity (VA)	Ocular findings	Treatment	Evolution	Follow-up (months)
Case 1	Male/39	Kidney transplantation 5 years earlier	3	OU	20/200 (RE) 20/25 (LE)	Kerato-uveitis (RE) Acute anterior uveitis(LE)	Topical acyclovir Topical dexamethasone Atropine 1%	Resolution and then recurrence of uveitis with granulomatous precipitates, patchy iris atrophy and distorted pupil. Final VA 20/25 OU.	24
Case 2	Male/42	Unremarkable	3	LE	20/20 (RE) 20/25 (LE)	Acute anterior uveitis	Topical acyclovir Topical dexamethasone Atropine 1%	Resolution of uveitis Final VA 20/20 (LE)	24
Case 3	Male/33	Unremarkable	3	LE	20/20 (RE) 20/30 (LE)	Acute anterior uveitis	Topical dexamethasone Atropine 1%	Resolution of uveitis Final VA 20/20 (LE)	12
Case 4	Female/40	Unremarkable	1	OU	Count fingers (RE) 20/200 (LE)	Geographic keratitis	Intravenous acyclovir and oral steroid	Resolution/Recurrence of keratitis with peripheral corneal scars Final VA 20/25 OU	18
Case 5	Male/35	Unremarkable	3	RE	20/40 (RE) 20/20 (LE)	Acute retinal necrosis	Intravenous acyclovir, intravitreal ganciclovir, and oral steroids	Resolution of inflammation Scarring in the peripheral retina Final VA 20/20 (RE)	18

#### Case 1

**Fig. 1:**
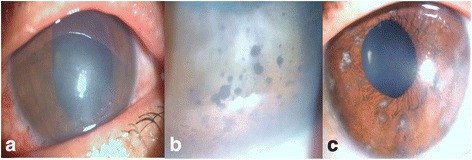
Slit-lamp photography of case 1. **a** Slit-lamp photograph showing disciform keratitis. **b** Slit-lamp photograph showing large pigmented corneal precipitates. **c** Slit-lamp photograph showing a patchy iris atrophy and a distorted pupil

A 39-year-old man, complained about a 3-day history of bilateral blurred vision. He had a past medical history of kidney transplantation 5 years ago and was receiving systemic immunosuppressive therapy. The patient was hospitalized for chickenpox and was treated with systemic antivirals 3 weeks prior to ocular symptoms onset. On ophthalmic examination, the patient’s best corrected visual acuity (BCVA) was 20/200 in the right eye (RE) and 20/25 in the left eye (LE). Slit-lamp examination showed stromal keratitis and few fine keratic precipitates in the RE (Fig. 1a) and 1+ anterior chamber cells in both eyes. Intraocular pressure was 22 mmHg in the RE and 20 mmHg in the LE. There were no vitreous cells, and results of dilated fundus examination were unremarkable in both eyes. The patient was diagnosed with keratouveitis in the RE and acute anterior uveitis in the LE secondary to chickenpox. He was treated with topical acyclovir five times a day, dexamethasone six times a day, beta blockers, and atropine 1% twice daily. He was lost to follow-up. One year later, he presented with a 1-week history of blurry vision bilaterally. On ophthalmic examination, BCVA was 20/200 in the RE and 20/30 in the LE. Slit-lamp examination showed “mutton-fat” pigmented keratic precipitates (Fig. 1b), 1+ anterior chamber cells, corneal scars, patchy iris atrophy with circumscribed depigmented spots of the iris, and distorted pupil (Fig. 1c) in both eyes. Intraocular pressure was 27 mmHg in the RE and 24 mmHg in the LE. Assessment of posterior segments revealed no abnormalities in both eyes. A diagnosis of recurrent granulomatous anterior uveitis was made. The patient was treated with topical dexamethasone six times a day, topical acyclovir five times a day, beta blockers, and atropine 1% twice daily. Intraocular inflammation gradually resolved, and visual acuity improved. Topical steroids and acyclovir were slowly tapered. Visual acuity improved to 20/25 in both eyes one month after presentation. No recurrence of intraocular inflammation was observed over a follow-up period of 24 months.

#### Case 2

A 42-year-old otherwise healthy man experienced redness and blurred vision in the LE 3 weeks after the onset of chickenpox. On examination, BCVA was 20/20 in the RE and 20/25 in the LE. His left pupil was small but reactive with no relative afferent pupillary defect. There were perilimbal injection, few fine keratic precipitates inferiorly, and 1+ anterior chamber cells in the LE. Intraocular pressure was 16 mmHg. There was no vitritis, and dilated fundus findings were normal. Examination results of the RE were normal. A diagnosis of unilateral acute non-granulomatous anterior uveitis secondary to varicella was made. The patient was treated by topical acyclovir five times a day, dexamethasone six times a day, and cycloplegic agent twice daily. The uveitis resolved within 2 weeks, and visual acuity improved to 20/20 in the LE. No recurrence of uveitis was observed over a follow-up period of 24 months.

#### Case 3

A 33-year-old man with no past medical history presented with a 3-day history of blurred vision and intense photophobia in the LE 3 weeks after the onset of mild chikenpox. On examination, BCVA was 20/20 in the RE and 20/30 in the LE. Slit-lamp examination of the LE showed perilimbal injection, fine keratic precipitates, and 2+ cells with 1+ flare in the anterior chamber. Intraocular pressure was 16 mmHg. There was no vitritis, and dilated fundus findings were normal. Examination results of the RE were normal. A diagnosis of unilateral acute non-granulomatous anterior uveitis secondary to varicella was made. The patient was treated by topical dexamethasone six times a day and cycloplegic agent twice daily. The uveitis resolved within 2 weeks, and visual acuity improved to 20/20 in the LE. No recurrence of uveitis was observed over a follow-up of 12 months.

#### Case 4

**Fig. 2:**
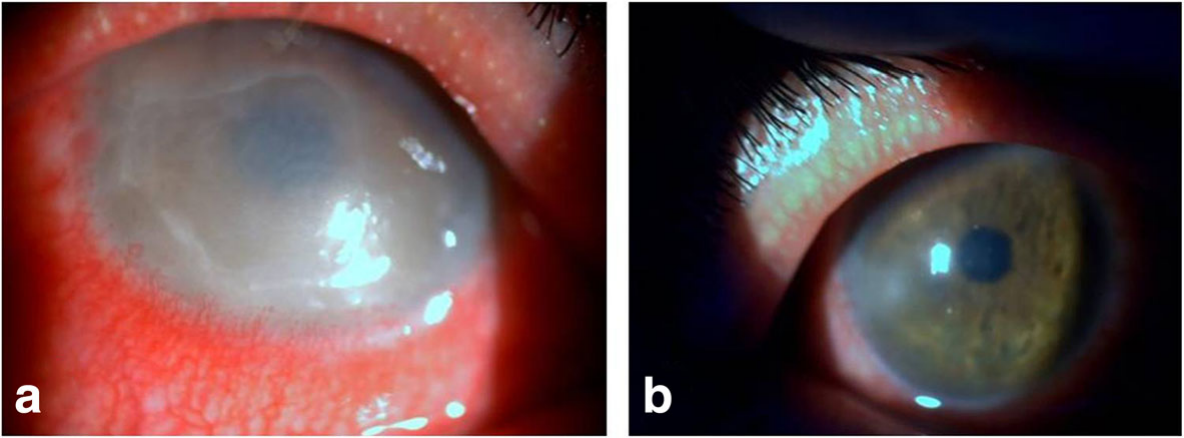
Slit-lamp photography of case 4. **a** Slit-lamp photograph showing a large geographic epithelial defect with stromal edema. **b** Slit-lamp photograph showing resolution of the corneal defect and corneal edema after treatment with antivirals and oral steroids

A 40-year-old otherwise healthy woman noticed pain, redness, watering, photophobia, and decreased vision in both eyes one week after the onset of severe chickenpox. On examination, BCVA was counting fingers in the RE and 20/200 in the LE. Slit-lamp examination showed perilimbal injection and a geographic corneal epithelial defect associated with diffuse stromal edema in both eyes (Fig. 2a). The anterior chamber was deep and quiet, and there were no vitritis or abnormal fundus findings.

A diagnosis of varicella keratitis was made. The patient was treated with systemic acyclovir (30 mg/kg/day). Oral steroids (0.5 mg/kg/day) were added 6 days after antiviral treatment, because of the severe associated stromal edema. The corneal defect resolved slowly within 2 weeks, and the medication was discontinued.

Three months later, the patient presented with complaints of decreased vision and eye pain bilaterally. On examination, BCVA was 20/35 in both eyes. Slit-lamp examination showed perilimbal injection, many small raised dendriform lesions, and marginal infiltrates in the cornea of both eyes. A diagnosis of recurrent varicella keratitis was made, and the patient was once again treated with systemic acyclovir (30 mg/kg/day) and oral steroids (0.5 mg/kg/day) 4 days later. Corneal lesions resolved within 2 weeks with residual peripheral corneal scars (Fig. 2b), and visual acuity improved to 20/25 in both eyes. No recurrence of keratitis was observed after 18 months of follow-up.

#### Case 5

A 35-year-old otherwise healthy man presented with a 1-week history of blurred vision in his RE. Medical history revealed mild chickenpox 3 weeks prior to his presentation, which resolved without complications. On ophthalmic examination, BCVA was 20/40 in the RE and 20/20 in the LE. There was no afferent pupillary defect. The intraocular pressure was 16 mmHg in both eyes. Minimal conjunctival hyperemia was present, and there were a few anterior chamber cells and 3+ vitreous cells and haze in the RE. Funduscopy revealed whitish retinal infiltration affecting the far periphery of the retina in one quadrant. Clinical examination results of the LE were normal.

A diagnosis of ARN syndrome secondary to chickenpox infection was made, and the patient was treated with intravenous acyclovir (10 mg/kg, three times a day). Seven days later, there was no improvement in the RE, and the patient was given intravitreal ganciclovir (2 mg). After a 10-day course of intravenous acyclovir, the patient received valacyclovir (1000 mg, three times daily). Seven days after initiation of oral antiviral therapy, he received oral prednisone (0.5 mg/kg/day for 1 week, followed by gradual tapering over 8 weeks). Systemic corticosteroids were prescribed because of significant vitritis. BCVA of the RE improved to 20/20, with gradual resolution of vitreous inflammatory reaction. The peripheral retinal lesion healed with a residual chorioretinal scar. No fellow eye involvement or recurrence of ARN was observed after an 18-month period of follow-up.

### Discussion

There have been only a few descriptions of chickenpox-related ocular complications in adults [1–7]. To the best of our knowledge, this series of five adults (seven eyes) with ocular involvement secondary to chickenpox is the largest reported to date. Similarly to previous reports, our results show that ocular complications of varicella may affect adult patients regardless of their immune status [1–7].

Previous data show that ocular disease may develop a few days to several weeks after the onset of skin rash [7, 8]. This interval ranged from 1 to 3 weeks in our patients. Ophthalmic involvement may result from a direct viral replication or from an immune reaction to the systemic viral infection [8].

Chickenpox ocular manifestations are diverse including conjunctivitis, keratitis, anterior uveitis, ARN, retinal vasculitis, chorioretinitis, optic neuritis, neuroretinitis, scleritis, and internal ophthalmoplegia or external ophthalmoplegia [7, 8]. As seen in our patients, ophthalmological involvement may be unilateral or bilateral [1, 5, 7, 8]. Ocular findings in our study included acute anterior uveitis in four eyes (67%), with associated stromal keratitis in one eye (17%), keratitis in two eyes (33%), and ARN syndrome in one eye (17%).

Consistent with our data on adults, anterior uveitis is one of the most frequently reported ocular manifestations of chickenpox in childhood. After the exanthema presentation, up to 25% of children with chickenpox were found to have acute anterior uveitis [12], but we are unaware of any published cases of isolated anterior uveitis in adult with varicella. The chickenpox-associated uveitis is usually characterized by fine keratic precipitates and occurs most often during the convalescent period [10, 13]. Fibrinous exudates may be seen in association with the precipitates, and in some cases, relapses with more severe iritis occur. Patchy iris atrophy (vitiligo iridis), appearing as small, round, depigmented areas in the iris stroma, similar to those seen in one of our patients, appears to be a typical feature of chickenpox-associated anterior uveitis [10]. A sector-like iris atrophy may also develop [9].

Acute anterior uveitis in children is usually mild and self-limiting, requiring only treatment with cycloplegic agents with or without topical steroids [8, 13]. However, data on management modalities of chikenpox-associated anterior uveitis in adults are lacking. Use of either topical or systemic antivirals may be left to the discretion of the treating ophthalmologist in mild cases. Antivirals should, nevertheless, be better considered in patients with more severe involvement without or with associated keratitis, as seen in case 1.

Isolated corneal involvement was observed in one patient in our study, as bilateral recurrent geographic epithelial ulceration. Various forms of keratitis has been previously described in association with chickenpox in adults and children including mild punctate staining of the cornea, dendriform keratitis, disciform keratitis, and necrotizing keratitis with ulceration [9]. Prompt diagnosis and management with topical or systemic antivirals are mandatory to avoid permanent visual impairment. Corticosteroids are required in cases with stromal involvement. Topical steroids are used in the absence of an associated epithelial defect. Otherwise, oral steroids may be prescribed [10, 11, 14, 15].

Although VZV is the most common causative agent of ARN syndrome [16], this clinical entity has been rarely described following chickenpox infection. The affected patients include healthy and immunocompromised adults, or children [1, 17].

Chickenpox-associated ARN syndrome seems to be milder than the “typical” ARN syndrome, with peripheral retinitis progressing at a slower pace, a more moderate inflammatory reaction in the vitreous and anterior chamber, a better visual acuity, and a rare occurrence of retinal detachment. Bilateral involvement is uncommon and has been previously described in only one immunocompromised patient [1]. Our patient with ARN was treated with systemic and intravitreal antiviral drugs. Adjunctive systemic corticosteroids were added only after stabilization of retinal lesions. The patient was then closely monitored for any exacerbation of intraocular inflammation or necrotizing retinitis [18]. The evolution was favorable with healing of retinitis lesions and gain of a normal visual acuity.

In conclusion, the spectrum of chickenpox-associated ocular complications in adults is wide. This case-series highlights the necessity for a systematic ocular examination including slit-lamp evaluation and dilated fundus examination in patients exhibiting any visual symptoms following a primary varicella. Early diagnosis and appropriate management are crucial to improve visual outcome.

## Abbreviations

ARN: Acute retinal necrosis
BCVA: Best corrected visual acuity
LE: Left eye
RE: Right eye
VZV: Varicella-zoster virus
